# Understanding the Causal Impact of Elevated Maternal Stress During Pregnancy: A Systematic Literature Review of Guinea Pig Models

**DOI:** 10.1101/2025.07.30.667555

**Published:** 2025-11-24

**Authors:** Rebecca L. Wilson, Caitlin Messiah, Adetola F. Louis-Jacques

**Affiliations:** 1 Center for Research in Perinatal Outcomes, University of Florida College of Medicine, Gainesville, Florida, USA 32610; 2 Department of Obstetrics and Gynecology, University of Florida College of Medicine, Gainesville, Florida, USA 32610

**Keywords:** Maternal stress, pregnancy, guinea pig, developmental programming, review

## Abstract

Increased maternal stress during pregnancy has been linked to numerous adverse pregnancy outcomes, including preterm birth and impaired fetal development. While clinical studies have established strong associations between maternal stress and adverse pregnancy outcomes, understanding the biological mechanisms is crucial for developing effective preventive intervention. Rodent models have provided valuable insights, however physiological differences from humans limit translational relevance. This systematic review evaluated and synthesized experimental models of increased maternal stress during pregnancy in guinea pigs. Guinea pigs share key pregnancy characteristics with humans, including relevant placental structure, maternal hormonal recognition of pregnancy and precocial offspring development. We identified 43 studies using various experimental approaches categorized into three main types: behavioral models, drug/substance exposure models, and physiological challenge models. These studies demonstrated significant fetal and offspring effects, particularly sex-specific neurodevelopmental outcomes. However, maternal physiological adaptations were often superficially characterized, focusing primarily on weight gain and occasional stress hormone measurements. Most studies began interventions during established pregnancy, potentially missing critical pre-pregnancy and early pregnancy periods. Overall, this review highlights the need for future studies to consider pre-conception interventions to better model human conditions and more comprehensively examine maternal adaptations.

## Introduction

Increased maternal stress, including psychological stress (example, anxiety or depression) and physical stressors (example, malnutrition or infection), during pregnancy has been linked to numerous adverse pregnancy outcomes in humans. Clinical studies have demonstrated associations between maternal stress and increased risk of preterm birth (Dole et al., 2003; Copper et al., 1996), low birth weight (Wadhwa et al., 2004) and preeclampsia (Zhang et al., 2013). Additionally, the impact of high maternal stress during pregnancy has been shown to have extensive effects on fetus and offspring health and development [1, 2].

The biological mechanisms underlying the associations between high maternal stress and adverse pregnancy outcome are complex but likely involve a number of key physiological mechanisms. Activation of the maternal hypothalamic-pituitary-adrenal (HPA) axis, resulting in elevated cortisol levels can affect placental function and fetal development [3, 4]. Maternal stress can trigger inflammatory responses and alter immune function, potentially compromising pregnancy maintenance [5]. Additionally, the timing of stress exposure appears particularly important, with different gestational periods showing varying susceptibility to stress-induced complications [6, 7]. While human studies have provided valuable insights into these relationships, animal models are essential for understanding the causal relationships between increased maternal stress, pregnancy outcome and long-term offspring health.

Rodent models are a cost-effective option for studying causal relationships and have been valuable in investigating potential mechanisms linking maternal stress to adverse pregnancy outcomes. Most consistent findings across rodent models are those relating to fetal growth and placental function [8–10]. Outcomes often reported include reduced litter size and litter weight and activation of the maternal HPA-axis that is associated with reduced circulating progesterone and alterations in placenta structure and functions. However, while human studies consistently show associations between maternal stress and increased risk of preterm birth [11], rodent models have produced variable results regarding gestational length [12]. In those that did report early delivery, gestational length was only minimally premature [13, 14]. The variability in pregnancy outcomes might reflect differences in stress protocols, timing of exposure, or species-specific responses to maternal stress [15].

The guinea pig (Cavia porcellus) has emerged as a particularly valuable model species for studying maternal-fetal interactions due to its similarities to human pregnancy, including comparable placental structure, relatively long gestation period, and advanced fetal development [16–18]. Guinea pigs, unlike rats and mice, exhibit similar hormonal profiles to humans during pregnancy, particularly in their production and regulation of progesterone, estrogen, and stress hormones [19, 20]. The maternal hypothalamic-pituitary-adrenal (HPA) axis undergoes comparable adaptations during pregnancy, including increased basal cortisol levels and altered stress responsiveness [21]. Importantly, like humans, but unlike most rodents, guinea pigs primarily produce cortisol as their main glucocorticoid, rather than corticosterone [22]. This is significant because cortisol plays a crucial role in maternal metabolic adaptation to pregnancy and the timing of parturition in both species [23, 24]. Additionally, the guinea pig’s relatively long gestation period (~65–70 days) allows for detailed study of temporal changes in maternal physiology across pregnancy, more closely matching the timeline of human gestational adaptation than shorter-gestation rodent models.

Over the past two decades, researchers have developed various experimental approaches to investigate the causal connections between increased prenatal maternal stress, pregnancy outcome and offspring developmental outcomes using guinea pig models (see references summarized in this review). These studies have encompassed a wide range of interventions, from psychological stressors to physiological challenges, providing insights into both the immediate and long-term consequences of prenatal stress exposure, particularly in offspring. The diversity of experimental approaches means multiple aspects of fetal and offspring development, including neurodevelopmental outcomes, cardiovascular function, and metabolic programming have been examined. This systematic review aimed to evaluate and synthesize the available literature on maternal stress during pregnancy using guinea pig models, focusing on experimental manipulations published between 2000 and present. We specifically examine the types of interventions employed, the timing of these interventions during pregnancy, and their effects on both maternal and offspring outcomes. This analysis will provide a critical overview of current knowledge in the field and identify gaps that require further investigation.

## Materials and Methods

### Eligibility Criteria

Studies included only those utilizing guinea pigs as a model species and focused on maternal experimental manipulation during pregnancy. There were no restrictions imposed on type of experimental manipulation but was specifically targeted to increased maternal stress, either psychologically or physiologically, during pregnancy. Given the heterogeneity of the observational strategies, a meta-analysis was not possible.

### Information Sources and Search

The search strategy and procedure was guided by the PRISMA statement [25]. Potential studies were located through electronic databases (Web of Science, Academic Search Premier and Scopus), as well as manual searches of references in review articles and relevant articles known by the authors. Limits included full text articles written in English and published in academic journals between 2000-present. The last search was performed in June 2025. Search terms and MeSH headings in the title, abstract, and index terms, were initially identified in Scopus and subsequent key words were used for the remaining databases (Appendix A). The search included the following: Maternal Stress or Stress; Pregnancy or Prenatal; Guinea Pig or Guinea Pigs.

### Data Collection

An independent search of the literature was performed in April 2025 and again in July 2025. Titles and abstracts were examined independently by two of the authors who documented reasons for excluding full text articles. Any differences between the two reviewers were clarified; a third reviewer resolved any disagreements. If an article appeared in duplicate from two or three of the databases, only the search containing the most relevant and useful information was included. For each eligible study, the following data was extracted: author, year; aim/hypothesis; study design; study outcome and preterm birth phenotype.

## Results

**Figure 1.**
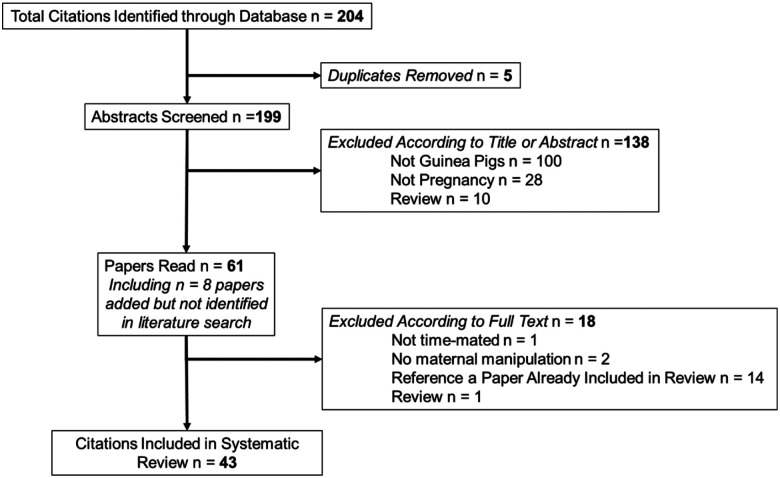
Flow diagram of the search strategy used in this review including the relevant number of papers at each point.

Figure 1 outlines the literature search and selection of studies. We identified 172 citations (10 duplicates) after searching Web of Science, Academic Search Premier and Scopus databases. A further 16 were added by authors after handsearching. After screening the title and abstract, 55 full text papers were read. Overall, 43 studies met the inclusion criteria and were included in the review (Table 1).

Most studies utilized timed pregnant guinea pigs with interventions occurring at specific gestational ages, typically between days 45–65 of pregnancy (term 65–70 days) [26]. One study began experimental manipulations from gestational day 1 [27] while only four studies [28–31] utilized a pre-conception experimental manipulation. The experimental designs generally included relatively small sample sizes, ranging from 4–16 animals per group, with most studies examining fetal and/or offspring outcomes. The majority of studies aimed to assess near-term fetal brain development [32–35] or neurodevelopmental outcomes in offspring [27, 36–53]. Additional aims included understanding fetal and/or offspring vascular function [54–56], fetal and/or neonatal lung development and function [57, 58], fetal liver damage [59], offspring auditory outcomes [60] and maternal impacts [61, 62].

Regarding pregnancy outcomes, most interventions did not significantly affect gestational length, though several models showed reduced fetal/offspring weights [31, 33, 50, 54, 56, 58, 59]. Fetal survival was often demonstrated except in cases of severe interventions such as infection models [63, 64]. Impacts to offspring were routinely sex-specific in nature [31, 34–36, 38, 39, 41–43, 46, 49]. For example, Crombie et al., [39] found that following maternal psychosomatic stress via strobe light in pregnancy, female offspring showed increased anxiety, decreased locomotion and increased freezing while male offspring showed hyperactivity behavior, increased locomotion and decreased fear behavior. When reported, maternal responses to experimental manipulations were generally well-tolerated with dams maintaining appropriate weight gain throughout pregnancy [34, 40, 41, 61, 62, 65] except with iron deficiency [29] and methadone treatment [47] where maternal weight gain was reduced. Increased maternal cortisol and stress responses were observed with strobe light [37, 45] and combined environmental stressors [34] but not with hyperthermia [62], chronic ethanol exposure [27, 33], iron deficiency [29] or endotoxin administration [40]. Adverse maternal outcomes were relatively rare, primarily occurring in infection models [64, 66].

The experimental manipulations to increase maternal stress used in these studies can be categorized into three main types. Stress and behavioral models [34–39, 41, 42, 49, 51, 60, 67]: maternal stress induced via strobe light exposure, maternal separation protocols, novel environment exposure, and social stress paradigms. Drug and substance exposure models: betamethasone or dexamethasone administration [43, 44, 46, 52, 53], ethanol exposure [27, 32, 33, 57, 58], methadone [47] and pharmacological intervention [65, 67]. Physiological models: uterine artery occlusion for growth restriction [45, 54], maternal nutrition manipulation [29–31, 61, 68], hypoxia exposure [50, 55, 56, 59], carbon monoxide [48], temperature [62] and infection/inflammation challenges [40, 63, 64, 66, 69].

### Stress and behavioral models

Stress and behavioral models in pregnant guinea pigs have been particularly well-characterized, with several research groups establishing reproducible protocols that demonstrate clear offspring effects. The most commonly used protocol involved exposure to strobe light stress, typically administered for 2-hour periods during specific gestational windows [36, 41]. This model induced a reliable maternal stress response, evidenced by elevated salivary cortisol levels, without causing pregnancy loss or significantly altering gestational length [37]. The timing of stress exposure appears critical, with different windows of exposure producing distinct offspring phenotypes, particularly in behavioral and neuroendocrine outcomes [41, 51]. The physiological basis for these behavioral changes has been extensively investigated, with studies revealing alterations in offspring HPA axis function [28, 37] and modifications in neurotransmitter systems [43].

More complex stress paradigms have also been developed, including chronic unpredictable stress protocols that better mirror human psychological stress [34, 35] and social environment manipulations [28, 49, 67]. [34] demonstrated that combining multiple stressors, including novel environment exposure, social stress, and intermittent food availability, created a more comprehensive model of chronic maternal stress. This approach showed that maternal HPA axis activation persisted throughout gestation, with increased basal cortisol levels evident by mid-gestation. The offspring effects of these various stress protocols were sex-specific, with males generally displaying increased anxiety-like behaviors and hyperactivity, while females showed more subtle behavioral alterations [39, 42, 49]. Importantly, these behavioral changes persisted into juvenile periods, suggesting permanent programming effects [37]. Some studies have attempted to ameliorate these stress-induced changes through postnatal interventions or environmental enrichment, though with varying success [35, 38].

### Drug and substance exposure models.

Drug and substance exposure models primarily focused on glucocorticoids and ethanol. Betamethasone and dexamethasone were studied because of their use to accelerate fetal lung maturation in cases of premature delivery [70, 71]. Early work by Dean et al., [46] showed that dexamethasone exposure during rapid brain growth altered offspring HPA function in a sex-specific manner, with increased resting cortisol in males but not females. Multiple studies examined both single and repeated courses of betamethasone, also administered at critical windows of brain development [43, 44]. Studies showed that maternal betamethasone administration affects offspring brain development, particularly myelination patterns and GABA receptor expression, with effects that persist into juvenile periods [43]. Additionally, the effects of single or repeated betamethasone exposure have been shown to affect HPA-axis function in both first and second-generation [52, 53]. These transgenerational effects show distinct sex-specific patterns and suggest that clinical use of antenatal steroids may have longer-lasting consequences than previously recognized.

Ethanol exposure models were also well-characterized, providing important insights into fetal alcohol spectrum disorders. These studies typically utilized chronic exposure protocols, with ethanol administered either through drinking water or direct administration [27, 33]. Key findings demonstrated that prenatal ethanol exposure affected multiple systems, including altered HPA axis function, modified glutamate signaling, and immune system development, particularly alveolar macrophage function [57].

Several other pharmacological interventions were studied and provided insights into programming effects of modulating the maternal HPA axis. Vartazarmian et al., [65] investigated prenatal selective serotonin reuptake inhibitor (fluoxetine) exposure and demonstrated altered pain thresholds in adult offspring despite no effects on pregnancy outcomes while Kaiser et al., [72] examined the effects of adrenocorticotropic hormone administration during pregnancy, finding increased aggressive behaviors and elevated cortisol levels in female offspring.

### Physiological models.

Physiological models focused primarily on three key areas: growth restriction (uterine artery ligation), hypoxia exposure, and infection/inflammation challenges. Growth restriction models produced fetal growth restriction while maintaining pregnancy viability, allowing investigation of both immediate and long-term consequences of restricted fetal growth [29, 30, 54]. Cumberland et al., [45] demonstrated that the combination of growth restriction and prenatal stress increased the risk of preterm labor, while growth restriction alone did not affect gestation length. These models have also shown impacts on fetal endocrine function, with growth restriction combined with prenatal stress resulting in decreased fetal circulating cortisol levels and altered allopregnanolone concentrations. Some studies have explored potential therapeutic interventions, such as N-acetylcysteine supplementation, showing promising results in ameliorating some adverse effects of growth restriction, particularly regarding vascular function and oxidative stress markers [54, 56].

Nutritional models have demonstrated diverse impacts of maternal dietary deficiencies and restrictions on fetal/offspring development. Models specifically targeting a particular nutrient deprivation (maternal vitamin C or iron deficiencies) indicated particular gestational or postnatal age effects. For example, maternal vitamin C deficiency caused transient intrauterine growth restriction at gestational day 45 which resolved by gestational day 56 [61]. Iron deficiency implemented from pre-conception through lactation resulted in elevated cortisol in offspring at postnatal day 24 that normalized by day 84, suggesting developmental programming effects on stress responsiveness that were correctable with postnatal nutritional rehabilitation [29]. Global nutritional restrictions provided insights into placental adaptations and fetal growth programming including placental transcriptome changes relating to DNA repair, apoptosis regulation, nutrient transport and IGF1 regulation [30] and sex-specific effects on hypoxia and oxidative stress markers in the fetal brain [31]. Collectively, these nutritional models demonstrate that different types and severities of maternal dietary restrictions produce distinct patterns of fetal adaptation, from transient growth effects with micronutrient deficiencies to more profound metabolic and transcriptional changes with global caloric restriction.

Infection and inflammation models provided crucial insights into mechanisms of preterm birth and fetal inflammatory responses. Studies using various pathogens, including E. coli [63], Brucella [64], and cytomegalovirus [66], have increased understanding of maternal-fetal transmission and fetal immune responses. These models showed that timing and severity of infection significantly impact outcomes, with early gestation infections often leading to pregnancy loss [64, 66], while later infections resulted in fetal inflammation without pregnancy termination [63]. The work by Hensel et al., [64] particularly demonstrated how maternal infection can lead to placental inflammation and fetal compromise, with specific patterns of inflammatory markers that parallel human responses.

Hypoxia models were used to understand fetal adaptations to reduced oxygen availability, with protocols ranging from chronic moderate hypoxia to more severe intermittent exposures and carbon monoxide exposure [48, 50, 55, 56, 59]. These studies revealed important insights into fetal cardiovascular adaptations, including changes in endothelial nitric oxide synthase expression and vascular remodeling, and redox imbalances in the neonatal brain. Fetal organs respond differently to hypoxic challenges, with tissue-specific alterations in oxidative stress markers and cellular adaptation mechanisms [59]. Whilst studies that extended the understanding of hypoxia effects beyond the fetal period and demonstrated long-term cardiovascular programming in female offspring [55] and mechanistic understanding of how prenatal hypoxia may predispose neuronal dysfunction in adulthood [50].

## Discussion

**Figure 2.**
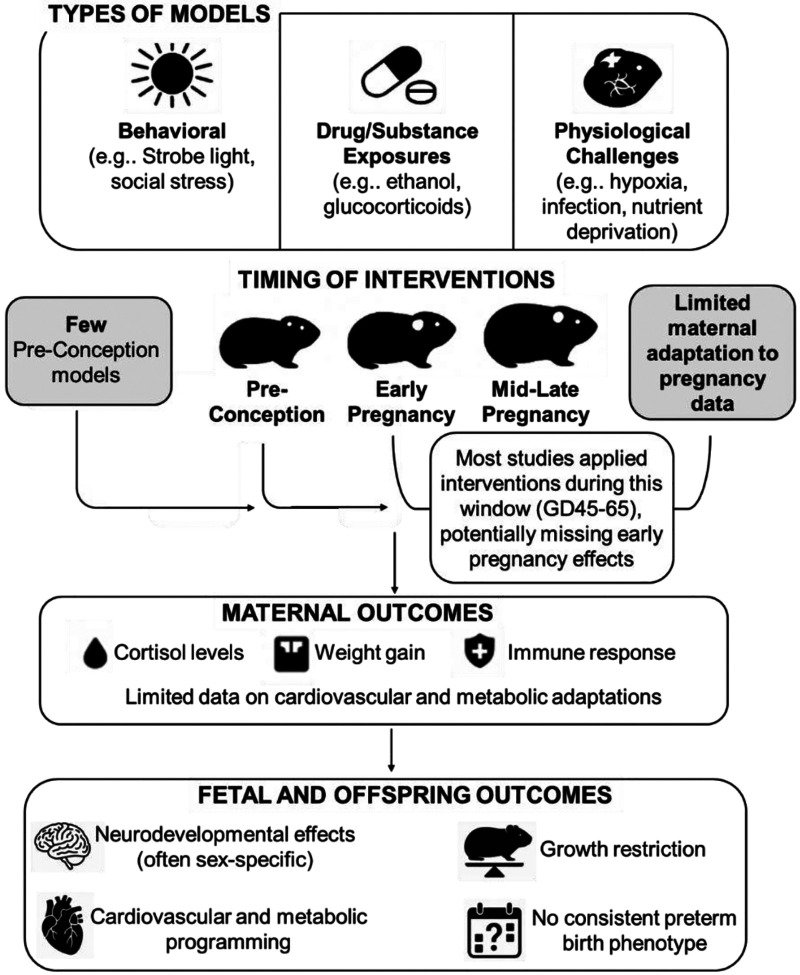
Summary of systematic review of the literature aimed to evaluate and synthesize the available literature on maternal stress during pregnancy using translational guinea pig models.

This systematic review aimed to evaluate and synthesize the available literature on maternal stress during pregnancy using translational guinea pig models: Summarized in Figure 2. Overall, analysis of the available literature revealed a predominant focus on fetal and offspring outcomes, with relatively limited examination of maternal physiological adaptations to various stressors. While experimental designs successfully demonstrated diverse methods of increasing maternal stress, from acute psychosocial stressors to chronic physiological challenges, maternal responses were often only superficially characterized through basic measures such as weight gain and occasional cortisol sampling. Even when maternal stress was confirmed through elevated cortisol levels or behavioral changes, the underlying maternal adaptations and potential compensatory mechanisms remained largely unexplored. This knowledge gap is particularly notable given that maternal physiological responses likely play a crucial role in mediating the well-documented offspring effects, which frequently showed sex-specific patterns in behavioral, endocrine, and developmental outcomes.

Of the studies that examined maternal responses, most focused on basic physiological markers such as weight gain and stress hormones. Maternal weight gain was generally maintained during stress interventions [34, 40, 41, 62], with reductions only observed in specific challenges like iron deficiency [29] and methadone treatment [47]. These findings parallel human studies where severe nutritional deficiencies or substance use affect maternal health [73, 74]. Maternal HPA axis activation was primarily assessed through cortisol measurements in blood or saliva. Elevated cortisol levels were observed following acute stressors such as strobe light exposure [37, 45] and combined environmental stressors [34]. Interestingly, several interventions including chronic ethanol exposure [27, 33] and iron deficiency [29] did not elicit increased cortisol responses, suggesting a possible adaptation of the maternal stress response system to chronic stress. Maternal adaptations to chronic maternal stress have also been observed in human pregnancies with chronic stress exposure [75]. These adaptations include changes in the HPA-axis function and cortisol patterns that attenuate stress reactivity and blood pressure responses [76]. Cardiovascular and metabolic adaptations have also been studied revealing differential hemodynamic responses and neuroendocrine reactivity [75]. Based on the literature reviewed here, the most severe maternal outcomes were observed in infection models [64, 66], particularly with high pathogen doses, mirroring the significant maternal morbidity associated with severe infections during human pregnancy [77, 78]. However, detailed characterization of maternal immune responses, cardiovascular adaptations, metabolic changes and behavioral modifications were largely absent from these studies. Overall leaving critical gaps in our understanding of how maternal systems adapt to maintain pregnancy despite significant stressors.

Despite extensive evidence linking maternal stress to preterm birth in human populations, the experimental models reviewed here showed little impact on gestational length, even with significant maternal stress manipulations. Cumberland et al., [45] demonstrated increased preterm labor and specifically when intrauterine growth restriction was combined with prenatal stress, whilst Maki et al., [31] reported preterm delivery in 3 of 18 nutrient restricted dams. These finding suggest that multiple ‘hits’ may be necessary to initiate mechanisms resulting in spontaneous preterm delivery and highlights a critical gap in our understanding of the mechanisms linking maternal stress to pregnancy termination. Studies identified in the literature search that did result in spontaneous preterm birth but were subsequently excluded because no maternal stress experimentation was utilized, were artificially induced through pharmacological means (aglepristone and oxytocin)[79–81]. Pharmacological induction of parturition was used as a tool to study prematurity effects on fetal and offspring development rather than to understand spontaneous preterm birth mechanisms. Even in infection models, which showed the highest rates of pregnancy loss [63, 64, 66], the focus remained primarily on fetal outcomes rather than examining the maternal inflammatory and endocrine pathways leading to pregnancy termination. This gap in mechanistic understanding may partly explain why current clinical interventions for preventing preterm birth remain largely ineffective [82, 83], as they target downstream processes rather than the fundamental response pathways that initiate premature labor.

On of the goals of this systematic review was to synthesize the available literature on maternal stress during pregnancy using guinea pig models because of the translational potential guinea pigs offer. Traditionally, rodent models (particularly rats and mice) have been extensively used to study pregnancy and parturition. However, their fundamental differences in maternal adaptation to pregnancy responses and parturition initiation mechanisms limit their translational relevance for understanding human pregnancy physiology. Unlike humans and guinea pigs, where progesterone remains elevated throughout gestation and labor occurs without systemic progesterone withdrawal [19, 84, 85], mice and rats require a sharp decline in circulating progesterone to initiate parturition [19]. This distinct endocrine profile means that stress-induced or inflammation-mediated preterm birth in these species may operate through mechanisms that are not necessarily relevant to human pregnancy. Additionally, humans and guinea pigs shift progesterone production from the ovary to the placenta during pregnancy [86] so that pregnancy maintenance is independent of the ovary [87]; this mechanism is not observed in mice and rats [88]. Placental establishment and micro-structure in the guinea pigs more closely mirrors humans than mice and rats and guinea pigs deliver precocial young making which offers advantages in studying fetal and offspring responses to various experimental approaches. Maternal stress and glucocorticoid responses are also similar between humans and guinea pigs with cortisol, as opposed to corticosterone in mice and rats, being the primary glucocorticoid [22]. However, despite the similarities between humans and guinea pigs, the studies reviewed here demonstrate that even guinea pig models have not fully replicated the stress-induced preterm birth phenomenon observed in human populations. This suggests that additional factors, perhaps related to the complexity of human psychosocial stress or the interaction of multiple physiological systems, may be necessary to fully model the pathways leading to stress-induced preterm birth.

Another limitation in guinea pig models included in this review is that experimental approaches often began during established pregnancy rather than before conception or during early pregnancy. In humans, many risk factors for adverse pregnancy outcome are present before pregnancy, including chronic medical conditions (diabetes, hypertension), previous reproductive history, and social determinants of health (poverty, stress, racism) [89–92]. Human studies demonstrate that chronic maternal stress and inflammation present before conception significantly increase preterm birth risk, with one systematic review showing a 2–3 fold increased risk in women with pre-pregnancy anxiety or depression [93]. For many of the studies presented here, various maternal insults - including stress [36, 41], infection [63, 64], growth restriction [54], and drug exposures [27, 33] - created significant fetal effects, however, maternal physiology and health outcomes were often not reported. It is possible that the lack of reports on maternal health outcomes was because there was no significant effect to report. Hence furthering our hypothesis that experimental protocols that begin during pregnancy fail to capture the complex interplay of pre-pregnancy and early pregnancy factors that epidemiological studies have shown contribute to adverse pregnancy outcomes in humans [94, 95]. It is our belief that to develop more clinically relevant models, experimental manipulations likely need to begin prior to conception to better reflect human conditions where pre-existing maternal conditions or early pregnancy events contribute to adverse pregnancy outcome risk. This represents an important gap in the current literature and an opportunity for future research directions that could better align animal models with human clinical presentations.

## Figures and Tables

**Table 1.** Summary of studies systematically reviewed.

Ref.	Aim/Hypothesis	Study Design	Outcome	Preterm Birth
Stress and Behavior Models				
[60] Morimoto, 2017. DOI: 10.1111/jog.13194	Aim: to record auditory brain response thresholds and latencies of neonatal guinea pigs with noise exposures *in utero* at different gestational periods to investigate the relations between exposure periods and postnatal hearing levels.	Pregnant dams (n=17) were exposed to the 4-kHz pure tone employed as noise for 4 h at one of the following times in pregnancy: Early: gestational day 27–28 Mid: gestational day 47–48 Late: gestational day not specified Control group were not exposed to noise Dams were allowed to deliver spontaneously. Neonates were assessed between postnatal day 1 and 28	No data on pregnancy outcome Noise exposure to pregnant dams during the early, mid-, and late gestational periods exhibited significant effects on auditory brain response thresholds in newborns.	Information not provided
[34] Emack, 2008. DOI: 10.1016/j.yhbeh.2008.02.025	Aim: assess the effects of an extended period of unpredictable maternal stress during pregnancy and the early postnatal period in order to more closely model low SES in an animal model. Aim: examine maternal pituitary–adrenocortical activity as well as growth, HPA function and locomotor behavior in juvenile male and female offspring.	Pregnant dams (n = 19) were exposed to one of four stressors every second day in a pseudo-randomized order from gestational day 32 to 66. The four stressors were: Novel Environment: Exposure to a strobe light in a darkened environment twice per. Social Stress: placed in the home cage of an unknown female twice per day. Forced Foraging: forced to forage for food that had been mixed in their bedding for a 24 h period Intermittent Food Availability: exposed to short bouts (10 h) of food withdrawal to elicit a behavioral disturbance, as the animals are cognizant that the other animals around them have been fed. Control dams (n = 21) were left undisturbed throughout gestation and postnatal period except for routine cage maintenance. After gestational day 66, dams were left undisturbed to deliver normally. Following birth, mothers were again exposed to a randomized stressor every second day from postnatal day 1 to 23, using the same paradigms as those described above. Food was available ad libitum to the offspring, in a size-restricted area of the cage that did not allow maternal entry.	No effect on maternal body weight during pregnancy, gestational age at birth, litter size or sex ratio. No significant difference in maternal salivary cortisol prior to experiment and 10 days into the chronic maternal stress. Higher maternal basal cortisol levels from gestational day 51 until gestational day 61. Tendency for increased maternal salivary cortisol on postnatal day 10 but no difference on postnatal day 20. No effect on birthweight in male or female offspring. Shorter body lengths in males Reduced postnatal weight gain between birth and postnatal day 25 in male offspring. Smaller head circumference but no difference in body length in male offspring at postnatal day 25. No differences in morphometric measures in female offspring at birth or on postnatal day 25.	No
[35] Emack, 2011. DOI: 10.1016/j.yhbeh.2011.08.008	Aim: examine HPA activity at various time-points following weaning in both male and female offspring of mothers who experienced chronic maternal stress. Aim: examine locomotor behavior and attention and determined whether peripubertal environmental enrichment can reverse the effects of chronic maternal stress.	This is a longitudinal follow-up of the Emack 2008 study and included the same animals as described previously Offspring at postnatal day 25 were either housed in standard housing or environmental enrichment in same sex groups. Standard housing: pair-housed in a clear plastic cage. Environmental enrichment: group housing (6 to 10 animals per cage) in a large pen with toys and boxes that were changed regularly. Outcomes in offspring were assessed between postnatal day 35 and 120	Minimal effects on HPA activity in offspring Significant long-term effects on offspring behavior that are age-dependent and sex-specific. Environmental enrichment modified HPA function and behavior independently of chronic maternal stress. ↑ basal HPA function in female offspring with chronic maternal stress and environmental enrichment.	No
[67] Kaiser, 2001. DOI: 10.1016/S0306-4530(01)00009-9	Aim: investigate the effects of an unstable social environment on the behavior and endocrine system of the male offspring.	Pregnant dams were living in groups of one adult male and five adult females. Dams either remained in a constant group composition throughout their whole pregnancy and lactation: stable social environment. Or the composition of the dams was regularly changed beginning with the onset of the 1st dam’s 2nd period of pregnancy: unstable social environment. Dams were allowed to deliver naturally. N = 28 male offspring were assessed between postnatal day 20 and 103.	Unstable social environment male offspring rested with bodily contact up to an older age. Unstable social environment male offspring displayed courtship behavior more often indicating a delayed behavioral development. Delayed development of the hypothalamo–pituitary–adrenocortical–axis and a ↓ activity of the sympathetic–adrenomedullary–system. No effect on testosterone.	Information not provided
[49] Von Engelhardt, 2015. DOI: 10.1186/1742-9994-12-S1-S13	Aim: study the effects of individual housing during the second half of pregnancy, with a focus on offspring development during the first five weeks after birth.	Pregnant dams were assigned to individual housing (n=8) or group housed (n=6), starting from gestational day 17 to 27. Initial housing: All animals together in large groups (14 females + 2 males per batch) until pregnancy. Individual housing: small enclosures with enrichment. Group housing: large enclosure containing 6 females together with enrichment. Dams were allowed to deliver naturally Offspring were studied the day after birth and postnatal day 7.	No effect on litter size or fetal sex ratios. No effect on birth weight or body length. ↓ growth in female offspring compared to male offspring from individual housed dams. ↑ cortisol response to social separation in female offspring ↓ cortisol response to social separation in male offspring	Cannot tell from experimental design.
[41] Kapoor, 2005. DOI: 10.1113/jphysiol.2005.090191	Aim: assess the effect of short bouts of moderate maternal psychological stress at very specific periods of fetal brain development on HPA axis activity, anxiety-related behavior and blood pressure in offspring.	Pregnant dams were exposed to strobe light for 2 h on gestational day 50, 51 and 52 (n =12) or gestational day 60, 61 and 62 (n =9). A control group of pregnant guinea pigs (n =12) was left undisturbed throughout gestation except for routine maintenance. Offspring were assessed between postnatal day 5 and 80	No effect on maternal body weight or weight gain during pregnancy There was no effect on gestational length or litter size. No effect on offspring birth weight or body weight in the pre-weaning period. ↓ body weight during the post-weaning period in offspring exposed to prenatal stress from gestational day 60 to 62. ↑ brain-to-body-weight ratios at postnatal day 70 in offspring exposed to prenatal stress from gestational day 50 to 52. ↑ basal plasma cortisol, ↓ plasma testosterone concentrations, and ↑ anxiety-related behavior in male offspring exposed to prenatal stress from gestational day 50 to 52. In contrast, maternal stress near term results in male offspring that exhibit ↑ adrenocortical in male offspring exposed to prenatal stress from gestational day 60 to 62.	No
[42] Kapoor, 2011. DOI: 10.1113/jphysiol.2010.200543	Hypothesis: decreased testosterone levels in male offspring born to mothers exposed to stress during the fetal brain growth spurt, at least in part, mediates the differences in behavior and endocrine activity exhibited by these animals. Hence, increasing plasma testosterone levels would ameliorate the differences in anxiety and attention-related behaviors and HPA axis activity.	Pregnant dams were exposed to the strobe light for 2 h on gestational day 50, 51 and 52 (n =15) or left undisturbed except for routine maintenance (n = 12). On postnatal day 75, male offspring were divided into four groups: control offspring with sham gonadectomy (n = 8), control offspring with gonadectomy plus testosterone replacement (n = 8), prenatal stress offspring with sham gonadectomy (n = 10) and prenatal stress offspring with gonadectomy plus testosterone replacement (n = 8). Male offspring were assessed between postnatal day 25 (pre surgery) and 85 (post surgery).	Prenatal stress affects locomotor activity, sensorimotor gating (an index of attention) and the acoustic startle reflex (an index of fear) in male offspring. ↓ plasma testosterone in adult prenatal stress male offspring. ↑ testosterone in gonadectomy and testosterone replacement prenatally stressed male offspring. Reversal of the behavioral effects of prenatal stress, but no change in HPA function.	
[28] Schopper, 2011. DOI: 10.1007/s00360-011-0591-1	Hypothesis: glucocorticoid levels should be elevated after stress exposure. Hypothesis: body weight should be attenuated in treatment but effects on reproductive performance are not necessarily expected.	Pregnant dams (n = 7) were exposed to strobe light twice daily (morning and afternoon) for 2 hours 7 days prior to conception, at conception and gestational days 7, 14, 28, 35 and 42. Pregnant control dams (n = 7) were left completely undisturbed in home cages during all stress periods.	All dams became pregnant No effect on duration of gestation, sex ratio, litter size, birth weight, total litter weight, total placental weight and gestational effort. Effect on hypothalamic–pituitary–adrenal (HPA) axis and the development of body weight.	No
[37] Bennett, 2015. DOI: 10.1159/000437302	Aim: assess the effect of prenatal stress on sustained changes in key hippocampal neurodevelopmental markers in guinea pig offspring at 21 days of age, together with its effects on postnatal growth and behavior.	Pregnant dams (n=12) allocated to the stress protocol were exposed to a strobe light for 2 h on gestational days 50, 55, 60, and 65. Control dams (n=12) remained undisturbed in normal cage. All dams were allowed to deliver spontaneously. Offspring were assessed between postnatal day 1 and 21	↑ salivary cortisol after all exposure time points. ↑ head circumference, ↑ abdominal circumference and ↑ nose-rump length on postnatal day 21, despite no difference in body weight from birth to 21. Deficits in key markers of mature oligodendrocyte development and reactive astrocyte expression. Indicators of ↑ anxiety and ↓ attentive (inquisitive) behaviors. No effect on basal neurosteroidogenic pathways and the systemic adrenocortical responsiveness (cortisol and DHEA) to handling.	No.
[36] Bennett, 2013. DOI: 10.1080/10253890.2017.1378637	Aim: examine the effect of prenatal maternal stress and the associated increase in glucocorticoid exposure on fetal brain development during key growth periods in gestation. Aim: investigate the effect of prenatal stress when allopregnanolone was administered during the last 8 days of gestation.	Pregnant dams were stressed by exposure to strobe light (75 J/10 sec) for 2 h on gestational day 50, 55, 60 and 65. Control pregnant dams were handled the same as stressed but were not exposed to strobe light. Allopregnanolone (10 mg/kg/day) was administered twice daily from gestational day 60 to 68. Pregnant dams were euthanized at gestational day 69 or on the second consecutive day of full pubic symphysis opening (>2 cm diameter).	Brain sparing and neuroprotective growth adaptations in female fetuses. ↓ expression of markers myelinating oligodendrocytes, reactive astrocytes and mature neurons in both CA1 region of the hippocampus and cerebral cortical white matter in male fetuses.	No.
[51] Bennett, 2016. 10.1016/j.psyneuen.2016.04.011DOI:	Aim: assess the effect of prenatal stress in pregnant guinea pigs beginning from 35 days gestation, 50 days gestation and 60 days gestation on markers of two main cell types in the growing brain which develop during early-mid and late in pregnancy. Aim: assess measures of offspring anxiety-like behavior and exploration following prolonged, transient stress in pregnancy	Pregnant dams were stressed by exposure to strobe light (75 J/10 sec) for 2 h according either of the following: Gestational day 35–65 (n=18): exposure beginning day 35 and repeated every 5 days until day 65 (7 incidents) Gestational day 50–65 (n=22): exposure beginning day 50 and repeated every 5 days until day 65 (4 incidents) Gestational day 60–65 (n=12): exposure on day 60 and 65 (2 incidents) Control dams were removed from their holding rooms and handled but not exposed to strobe light.	↑ maternal salivary cortisol after stress exposure from gestational day 50 until 65, but not at timepoints before gestational day 50. No effect on gestation length ↓ birthweight of pups with the longest stress exposure (gestational day 35–65). Sex-specific alterations in brain cell types, behavior and neurosteroid ontogenesis in offspring.	No.
[39] Crombie, 2021a. DOI: 10.1016/j.psyneuen.2021.105423	Aim: examine the effect of prenatal stress, postnatal stress, and the combination of both stressors on outcomes in guinea pig offspring at an age equivalent to human childhood. Aim: examine the changes to progression of the oligodendroglial lineage leading to the disruption of mature myelin, and important components of the GABAergic and glutamatergic pathways in stress-induced neurological dysfunction.	Pregnant dams were assigned one of 4 groups: Control (n = 16), Prenatal Stress Only (n = 14), Postnatal Stress Only (n = 18) and Prenatal + Postnatal Stress (n = 15). Prenatal stress was induced by taking pregnant dams within their individual home cages and placing them in a dark, temperature-controlled room, fitted with a strobe light to induce psychosomatic stress with the flashing light (75 joules). Strobe light exposure occurred for 2 h on gestational day 50, 55, 60 and 65. Postnatal stress in offspring was caused by maternal separation for 2 h/day from postnatal day 1 to 7. Control dams and offspring groups were handled at the same time each day without causing stress. Dams were allowed to spontaneous delivered at term. Behavioral outcomes were assessed on postnatal day 27. Offspring were euthanized on postnatal day 30	Prenatal stress only = hyperactivity in male offspring and anxiety-like behavior in female offspring. Disrupted oligodendrocyte expression, myelination deficits, disturbed GABAergic components and glutamatergic imbalances. Postnatal stress only = increased neonatal cortisol, but this exposure alone did not result in adverse changes in behavior or deficits in neurodevelopment. Adverse effects of prenatal stress exposure were ameliorated when followed by a stressful postnatal environment, particularly in males.	No
[38] Crombie, 2021b. DOI: 10.1016/j.psyneuen.2020.105060	Aim: examine the effects of prenatal stress on components of the oligodendrocyte lineage to identify the key processes that are disrupted and to determine if postnatal therapies directed at ameliorating white matter deficits also improve behavioral outcomes.	Pregnant dams (n = 41) within their individual home cages, were placed in a dark, temperature-controlled room, fitted with a strobe light to induce psychosomatic stress with the flashing light (75 joules). Strobe light exposure occurred for 2 h on gestational day 50, 55, 60 and 65. Control dams (n = 49) were handled at the same time each day without causing stress. Offspring were spontaneous delivered at term. Offspring were then randomly allocated to receive either vehicle, oral ganaxolone (5 mg/kg twice daily) or Emapunil (0.3 mg/kg once daily) on postnatal days 1 to 7. Tissue collection was conducted on postnatal day 30.	Consistent with previous studies: Bennett et al., 2016, Crombie et al., 2021. Disrupted oligodendrocyte maturation lineage, translation of MBP mRNA-to-protein, neurogenesis and ↓ amount of mature myelin. ↑ anxiety, ↓ locomotion and ↑ freezing behavior in female offspring hyperactivity behaviors, ↑ locomotion and ↓ in fear behavior in male offspring. Postnatal ganaxolone and emapunil treatment restores the behavioral phenotype in female offspring only. Emapunil treatment restored mature myelin levels in both sexes, and reversed disruptions to the oligodendrocyte lineage in female offspring, an effect not seen with ganaxolone treatment.	No
Drug and substance exposure models				
[46] Dean, 2001. DOI: 10.1159/000054636	Hypothesis: exposure to synthetic glucocorticoid during rapid brain growth in fetal guinea pigs modifies hypothalamo-pituitary-adrenal (HPA) function after birth, and that this involves changes in central corticosteroid receptor regulation.	Pregnant dams were injected with dexamethasone (1 mg/kg; n = 11) or vehicle (n = 10) on gestational days 50 and 51 of gestation (term = 68 days). Dams were then left undisturbed and allowed to deliver naturally. Offspring remained with their mothers until postnatal day 18. At this time offspring were either euthanized instantly on removal from the maternal cage or immediately after 30-min isolation from the mother.	No effect on litter size or on birth weight ↑ in gestation length altered body and organ (brain, heart, adrenal) growth. ↑ resting plasma cortisol concentrations in young male, but not female offspring Elevated basal cortisol levels were not increased further by isolation. ↓ hippocampal glucocorticoid receptor (GR) mRNA in female offspring ↑ GR mRNA levels in male offspring Mineralocorticoid receptor mRNA in the limbic system and GR mRNA levels in the pars distalis were unaffected. ↓ Proopiomelanocortin mRNA in the male offspring pars intermedia.	No. Increased gestation.
[43] Owen, 2006. DOI: 10.1113/jphysiol.2006.122887	Hypothesis: that repeated prenatal glucocorticoid exposure during critical periods of fetal brain development would impact open-field behavior in juvenile offspring. Hypothesis: that alterations in N-methyl-D-aspartate receptor expression may underlie behavioral changes observed in these juvenile offspring.	Pregnant dams were injected with betamethasone (1 mg/kg; n =13) on gestational day 40/41, 50/51 and 60/61. Control pregnant dams (n = 15) received a sham saline injection at same timepoints. Offspring were assessed at postnatal day 10.	No effect on litter size and birthweight. No differences in pup weights or any organ weights at postnatal day 10. ↑ activity in an open-field in female offspring. ↓ NR1 subunit mRNA levels in CA1/2 and CA3 regions of the hippocampus in female offspring.	Information not provided
[52] Iqbal, 2012. DOI: 10.1210/en.2012-1054	Aim: investigate whether the effects of prenatal synthetic glucocorticoid exposure on HPA function and behaviors are present in second-generation (F2) offspring.	Pregnant dams (F0) were injected with three courses (each course consisted of two injections, 24 h apart) of either betamethasone (1 mg/kg; n=8) or vehicle (n=8) on gestational day 40/41, 50/51, and 60/61. Dams were allowed to deliver and offspring (F1) weaned at postnatal day 25. F1 females were bred with untreated control males and left undisturbed during pregnancy and delivery, apart from routine cage maintenance. F2 offspring were assess as juveniles and at adulthood	No effect on gestation length or birth weight (published in Dunn et al., 2010. PMID: 20064858) ↓ stress responsiveness and basal cortisol regulation in male and female F2 offspring Alterations in molecular regulation of the HPA axis at the level of the hippocampus and anterior pituitary. ↓ locomotor activity in a novel environment in the juvenile F2 males	No
[53] Moisiadis, 2018; DOI: 10.1210/en.2018-00666	Aim: assess whether antenatal exposure to a single course of synthetic glucocorticoids affects male F2 offspring and alters stress-associated behaviors, and whether these effects persist into adulthood.	Pregnant dams were injected (2 injections, 24 h apart) with either betamethasone (1 mg/kg; n =14) or vehicle (n=14) on gestational day 50/51). Dams were left to deliver F1 offspring undisturbed. F1 females were bred with untreated control males and left undisturbed during pregnancy and delivery, apart from routine cage maintenance. Offspring were assess as juveniles and at adulthood	No effect on gestation length in the F0-F1 pregnancy Strongest effect of synthetic glucocorticoid exposure occurred in adult F1 offspring with fewer effects in the F2 offspring. ↑ HPA stress response in F1 females ↓ HPA responsiveness in F1 males ↓ locomotor activity and prepulse in adult F1 offspring. ↓ HPA response in female F2 adult offspring associated with molecular changes in the anterior pituitary No behavioral changes were observed in F2 animals.	No
[44] Sasaki, 2021. DOI: 10.1038/s41398-020-01186-6	Aim: examine parallel genome-wide methylation profiles from the hippocampus and whole blood of juvenile guinea pig females subjected to maternal exposure to synthetic glucocorticoids. Hypothesis: that prenatal synthetic glucocorticoid administration would alter DNA methylation modifications in offspring hippocampus and whole blood.	Pregnant dams were injected with betamethasone (1 mg/kg; n =6) on gestational day 40/41, 50/51 and 60/61. Control pregnant dams (n = 6) received a sham saline injection at same timepoints. Offspring were assessed at postnatal day 14.	Over 90% of the common methylation signatures associated with synthetic glucocorticoid exposure showed the same directionality of change in methylation. Among differentially methylated genes, 134 were modified in both hippocampus and blood, of which 61 showed methylation changes at identical CpG sites. Gene pathway analyses indicated that prenatal synthetic glucocorticoid exposure alters the methylation status of gene clusters involved in brain development.	No
[27] Iqbal, 2005. DOI: 10.1210/en.2012-1054	Aim: determine the effects of chronic prenatal ethanol exposure on maternal cortisol concentration during pregnancy. Hypothesis: chronic prenatal ethanol exposure causes long-lasting changes in glucocorticoid signaling in postnatal offspring.	Pregnant dams were administered, as two equally divided doses separated by 2 h from gestational day 2 to 67, ethanol or isocaloric sucrose with pair-feeding to an ethanol-treated animal or isovolumetric water with ad lib access to food and water or untreated with ad lib access to food and water. Blood ethanol concentration was assessed on gestational day 57. Dams were allowed to deliver naturally. Offspring were assessed at adulthood.	No effect on the length of gestation, average litter size, or the distribution of male and female offspring. Perinatal death occurred four times in each of the ethanol and sucrose treatment groups, and two times in the water treatment group. No effect on saliva cortisol concentration above that induced by pregnancy. No effect on stimulated glutamate or GABA release Selectively prevented dexamethasone-mediated suppression of stimulated glutamate release. ↓ expression of mineralocorticoid, but not glucocorticoid, receptor mRNA in the hippocampus of offspring.	No
[57] Gauthier, 2005. DOI: 10.1203/01.PDR.0000149108.44	Aim: examine the effects of chronic in utero ethanol exposure on glutathione availability in the premature lung and the resulting impact on fetal alveolar macrophage function. Aim: determine whether glutathione supplements, in vitro or in vivo, would maintain or restore fetal alveolar macrophage function.	Pregnant dams on gestational day ~35 were provided ethanol or no ethanol in the drinking water with incremental increases up to 4% ethanol till gestational day ~40. Control dams were pair-fed solid diet to match ethanol dams intake. Where appropriate, the glutathione precursor S-adenosyl-methionine (1.0 mg/mL) was added to the drinking water containing the ethanol. Dams were euthanized on gestational day 55.	Impaired macrophage function and ↓ glutathione availability in the fetal lung with ethanol exposure. Maternal glutathione supplementation protected the developing macrophage from apoptosis and dysfunction.	N/A
[58] Xiao-Du, 2007. DOI: 10.1111/j.1530-	Hypothesis: dysfunction of the neonatal alveolar macrophage exposed to ethanol in utero would persist at term gestation. Aim: evaluate whether in utero ethanol caused alveolar macrophage dysfunction at term gestation. Aim: evaluate whether glutathione availability could protect the term alveolar macrophage from ethanol-induced dysfunction.	Same ethanol protocol as Gauthier 2005. The dams were allowed to proceed until spontaneous term delivery. Where appropriate, the glutathione precursor S-adenosyl-methionine (10 mM) was added to the drinking water to the Control or ethanol groups.	↓ body weight in ethanol exposed offspring ↑ body weight in ethanol and S-adenosyl-methionine exposed offspring. No difference in body weight with S-adenosyl-methionine alone. Ethanol-induced alveolar macrophage dysfunction in phagocytosis. ↑ apoptosis in the term alveolar macrophage.	No
[33] Hewitt, 2010. DOI: 10.1016/j.ntt.2009.12.002	Aim: tested the hypothesis that chronic ethanol exposure, via chronic maternal ethanol administration, increases CYP2E1 expression and HPA axis activity in the maternal–fetal unit during the third-trimester-equivalent of gestation.	Pregnant dams from gestational day 2 were exposed to ethanol (n = 12) or isocaloric-sucrose/pair-feeding (n = 12). Ethanol group: oral administration of 4 g ethanol/kg maternal body weight as an aqueous ethanol solution (30% v/v, prepared in tap water) and had ad libitum access to food. Pregnant dams were euthanized at 2 h after the last divided dose of ethanol or sucrose at gestational day 45, 55 or 65.	↑ maternal and fetal blood ethanol content. ↓ fetal hippocampal weight. no effect on fetal body weight. No apparent effect on the HPA axis in the mother or the fetus: no change in maternal or fetal plasma free cortisol and ACTH concentration. ↑ CYP2E1 activity in the mitochondrial and microsomal fractions of the maternal liver. ↑ in cytochrome P450 2E1 activity in maternal and fetal liver at gestational day 65.	N/A
[32] Hewitt, 2014. DOI:	Aim: test the hypothesis that the non-dominant glucocorticoid, corticosterone, was elevated by chronic ethanol exposure.	Same animals as Hewitt 20210	Augmented maternal plasma corticosterone concentration. ↑ fetal plasma corticosterone concentration, but not cortisol concentration at gestational day 65.	N/A
[47] Safa, 2021. DOI: 10.3389/fphar.2020.613328	Aim: develop methadone treated pregnant dams as a physiologically more suitable model, enabling detection of robust spontaneous neonatal withdrawal. Aim: show that the opioid antagonist 6BN can prevent spontaneous withdrawal behaviors after birth when co-administered with a prenatal opioid, and it can do so with high potency.	Pregnant dams on gestational day ~50 were given a daily single injection of saline (n = 7), methadone (n = 7), or methadone+6BN (n = 7) for an average of 15 ± 3 injections before birth at gestational day ~65. Pups were behaviorally tested (locomotion and vocalization) at 48 h after birth.	↓ maternal weight gain with increased MTD. ↑ offspring mortality at the higher MTD doses. No difference in offspring birthweight at doses 5–10 mg/kg MTD. ↑ offspring birthweight at 2 mg/kg MTD. ↑ offspring plasma cortisol with MTD. 6BN prevented MTD cortisol changes. MTD exposure aggravates classic maternal separation behaviors, including locomotor and vocal behaviors. 6BN, when delivered together with the agonist, can prevent dependence-related behaviors in offspring.	No
[72] Kaiser, 2000. DOI: 10.1016/S0031-9384(00)00248-1	Aim: elucidate the effects of adrenocorticotropic hormone application during pregnancy on the female offsprings’ endocrine status and behavior.	Pregnant dams (n=12) were treated with Adrenocorticotropic hormone (20 IU) or placebo on gestational day 30, 37 and 44 during two consecutive pregnancies so that they either received ACTH during the first and placebo during the second pregnancy or vice-versa. Dams were allowed to deliver naturally. Female offspring were studied from postnatal day 41 to 80.	↑ urine spray, a defensive aggressive behavioral pattern. No indications of a behavioral masculinization ↑ serum cortisol concentrations from postnatal day 62.	Information not provided
[65] Vartazarmian, 2005. DOI: 10.1007/s00213-004-2003-7	Aim: study the effects of prenatal fluoxetine (selective serotonin reuptake inhibitor) exposure on pregnancy characteristics, weight gain and two serotonin-modulated behaviors (i.e. thermal pain threshold and prepulse inhibition (PPI) of acoustic startle) in the adult offspring.	Fluoxetine (7 mg/kg per day) or vehicle was provided to pregnant days via osmotic pump from gestational day 1. At birth, all pups were cross-fostered with surrogate untreated dams. Offspring were weaned at 2 weeks of age and behaviorally tested at 9 weeks of age.	No effect on dam body weight at gestational day 0, 21 and 42. No effect on gestation length, live litter size or number of stillborns. No effect on offspring body weight at 2 or 9 weeks of age ↓ thermal pain thresholds in offspring at adulthood No effect on pre-pulse inhibition of startle in offspring at adulthood.	No
Physiological Models				
[54] Krause, 2019. DOI: 10.1111/apha.13328	Aim: study the endothelial function in carotid and femoral arteries, as well as the eNOS mRNA levels and Nos3 promoter DNA methylation pattern in aortic endothelial cells from fetuses and adult offspring from an FGR guinea pig model. Aim: assess effects of an antenatal antioxidant treatment on the endothelial function and vascular properties.	Pregnant dams (n=26) on gestational day 35 of pregnancy underwent uterine artery occlusion (FGR) or sham. Half of the FGR dams (n=8) were treated N-acetyl cysteine 12 (500 mg/day) in the drinking water. At gestational day 60 to 62 part of the control (n = 4), FGR (n = 4) and FGR/NAC (n = 4) dams were euthanized. Remaining sows [control (n = 5), FGR (n = 5) and FGR/NAC (n = 4)] were allowed to deliver and their offspring [control (n = 10), FGR (n = 10) and FGR/NAC (n = 8)] followed up until adult age.	↑ gestational age at birth in the FGR group. ↓ birthweight in the FGR group. Premature and permanent impaired vascular function. Antenatal maternal treatment with N-acetyl cysteine was able to prevent most of the effects of FGR.	No
[45] Cumberland, 2017. DOI: 10.1017/S2040174417000307	Hypothesis: intrauterine growth restriction in combination with prenatal stress will have a cumulative effect on allopregnanolone concentrations and the development of vulnerable brain regions in the limbic system of the fetus. Hypothesis: males will have a heightened vulnerability to potentiation by intrauterine growth restriction and prenatal stress with greater deficits compared with females. Aim: determine the effects on placental and plasma allopregnanolone concentrations. Aim: investigate the effects on myelination, mature neurons, GABA synthetic enzyme expression and levels of activated astrocytes.	Pregnant dams (n = 25) underwent surgery on gestational day 32 for uterine artery occlusion or sham. Uterine artery occlusion dams were further allocated to either control (n=6) or prenatal stress (n=10) that commenced on gestational day 40 and was repeated on 45, 50, 55, 60 and 65. Stress was induced by exposure to strobe light for 2 h on gestational day 40, 45, 50, 55, 60 and 65. Dams were euthanized on gestational day 69 or on determination that the pubic symphysis had begun separation and had reached >2 cm for 2 consecutive days.	↑ preterm labor with intrauterine growth restriction and prenatal stress. No effect on gestation length with just intrauterine growth restriction. No differences in litter sizes. ↓ fetal circulating cortisol with intrauterine growth restriction and prenatal stress. ↑ maternal salivary post-time stress exposure but no pre-exposure. ↓ circulating allopregnanolone levels with intrauterine growth restriction and prenatal stress. No effect on brain and placental allopregnanolone synthesis.	Yes.
[61] Schjoldager, 2015. DOI 10.1007/s00394-	Aim: investigate the impact of prolonged maternal vitamin C deficiency on maternal, fetal and placental weight parameters and fetal and placental vitamin C status at two different gestational stages during the second half of gestation.	Pregnant dams (n = 4/5 per group) from gestational day 6 to 10 were provided a sufficient (918 mg/kg feed) or deficient (100 mg/kg feed) vitamin C diet. Dams were euthanized at either gestational day 45 or 56.	No signs of pregnancy associated diseases. No difference in overall weight gain. Transient intrauterine growth retardation of the fetuses and their corresponding placentas at gestational day 45. No difference in fetal or placenta weight by gestational day 56.	N/A
[29] Shero, 2018. DOI: 10.1016/j.nutres.2018.03.017	Hypothesis: maternal iron deficiency (MID) elevates offspring serum cortisol, a biomarker of stress, during childhood and possibly at mature age. Aim: determine if MID during pregnancy and lactation had an impact on the cortisol secretion in the offspring.	Pregnant dams were assigned to an iron sufficient (114 mg/kg iron; n = 26) or iron-deficient (11.7 mg/kg iron; n = 12) diet. Diets were provided for 3 weeks prior to mating through pregnancy and lactation. Dams were allowed to deliver naturally. At postnatal day 9 offspring were weaned onto iron sufficient diets. The duration of the entire study was conducted in a 1-year timeline.	↓ estimated gestational weight gain per day in dams. ↓ hematocrit in dams over the 3 trimesters. No effect on circulating cortisol in dams ↓ increase of weight in offspring after weaning. ↑ circulating cortisol in offspring at postnatal day 24 which normalized by postnatal day 84	Information not provided
[30] Wilson, 2025. DOI: 10.1007/s43032-024-01769-4	Aim: understand the transcriptional changes within the placenta associated with placental insufficiency that occur prior to/at initiation of fetal growth restriction, and the impact of short-term *hIGF1* nanoparticle treatment.	Pregnant dams were assigned either an ad libitum Control diet (n = 7) or nutrient restricted diet (70% food intake diet based on kilogram of body weight of control; n = 12) from 4 weeks prior to conception. At gestational day 30 a subset of nutrient restricted dams (n = 7) underwent intra-placental *hIGF1* gene therapy treatment. Remaining nutrient restricted and Control dams received a PBS sham injection. Dams were euthanized 5 days after intra-placental injection.	Maternal nutrient restriction increased expression of genes associated with DNA repair, apoptosis regulation and enzyme inhibition and decreased expression of genes associated with nutrient transport, ion channels and IGF1 regulation in the placenta. *hIGF1* gene therapy treatment increased expression of genes associated with transmembrane transport and decreased expression of genes associated with lipid biosynthesis and hormone metabolism compared to sham treated.	N/A
[31] Maki, 2019. DOI: 10.1159/000506939	Aim: determined whether maternal nutrient restriction (MNR) in guinea pigs leading to fetal growth restriction (FGR) impacts markers for brain hypoxia and oxidative stress.	Pregnant dams were assigned either an ad libitum Control diet (n = 12) or nutrient restricted diet (70% food intake diet based on kilogram of body weight of control; n = 18) from 4 weeks prior to conception n until mid-pregnancy increasing to 90% thereafter. Dams were euthanized at gestational day 60–61.	↓ total fetal, brain and liver weights Unchanged total brain cell count/mm^2^ ↑ hydroxy-probe 1 positive cells in growth restricted male fetuses Variable ↑ in brain oxidative stress biomarker expression with growth restriction	Preterm delivery was reported in 3 of 18 MNR dams
[62] Michel, 2011. DOI: 10.1016/j.ygcen.2011.02.007	Aim: examine the influence of distinct (neutral versus cool) ambient temperatures on baseline and stress cortisol levels in pregnant dams. Aim: assess the impact of experimental treatment on reproductive output and the offspring 2 month after birth.	A “cool” mean temperature regime at 15 °C (n = 12), and a “neutral” mean temperature regime at 22 °C (n = 12) was employed starting between 0 and 20 days gestation. Number, sex and morphological characteristics the pups were recorded as soon as parturition was observed. Offspring were separated from dams 7 days after birth. Following separation, the offspring were maintained under stable thermal conditions. Offspring were assessed 2 months after birth.	No effect on dam weight gain and food consumption during pregnancy. Irrespective of the experimental ambient temperature and the time of the day, the positions adopted by guinea pigs inside their box did not produce any clear pattern ↓ activity of dams in cooler temperature. No effect of temperature on baseline cortisol. ↑ stress response following handling in dams exposed to cool temperature. Thermal treatment did not influence parturition date, the proportion of stillborns, mean litter size, mean litter mass or sex ratio Thermal treatment did not influence the characteristics of the offspring: body mass, body size, body condition or postnatal survival. ↑ amplitude of the stress response in the offspring born from the cool regime dams.	No.
[55] Paz, 2025. DOI: 10.1016/j.lfs.2024.123282	Aim: evaluate cardiac programming due to gestational hypoxia in female offspring. Aim: assess long-term cardiovascular risk	Pregnant dams at gestational day 30 were subjected to simulated conditions of normoxia (720 Torr; n = 6) or hypoxia (470 Torr; n = 6) in a hypobaric chamber until the day of delivery (~70 days of gestation). Dams delivered naturally. Offspring were assessed 1 year after birth.	No effect on offspring birth weight and litter size or body weight gain from birth until 28 days. ↑ heart rate in female offspring throughout the entire postnatal period ↑ pulsatility index throughout postnatal life Hemodynamic alterations, cardiovascular remodeling, endothelial dysfunction, and increased oxidative stress.	No.
[50] Figueroa, 2025. DOI:	Aim: describe the effect of gestational hypoxia on redox balance and apoptosis cell death mechanisms in the prefrontal cortex of guinea pigs.	Pregnant dams at gestational day 30 were introduced to a hypobaric chamber in conditions of normoxia (n = 5, controls, 720 torrs) or Hypoxia (n = 5, 470 torrs) until delivery. Dams delivered naturally. Offspring were euthanized at birth.	↓ biparietal diameter from gestational day 30 and abdominal circumference from gestational day 40 ↑ Cerebroplacental Ratio and ↓ placental biometry measures near term ↓ birthweight ↓ antioxidant sources and ↑ pro-oxidant sources in the prefrontal cortex, resulting in brain oxidative damage	Information not provided
[56] Thompson, 2005. DOI: 10.1203/PDR.0b013e31818d6ad0	Aim: determine the effect of chronic hypoxia on the protein expression of NOS3 in the fetal guinea pig heart.	Pregnant dams were housed in either normoxia (n=5) or hypoxia (12% O_2_) chambers for 14 (n = 5) or 28 (n = 5) days prior to euthanasia. At gestational day ~60 dams were euthanized.	No effect on gestational age or litter size. ↓ fetal body weight with hypoxia. ↓ expression of NOS3 protein in the feta heart.	N/A
[59] Hashimoto, 2012. DOI: 10.1177/1933719112440052	Aim: quantitate the effect of chronic hypoxia on the fetal liver in generation of oxidative stress Aim: test the protective effect of maternal administration of an antioxidant, N-acetylcysteine (NAC), against the fetal liver damage in the hypoxic fetus.	Pregnant dams were placed in either a hypoxic chamber (10.5% O_2_ for 14 days prior to fetal excision, n=6) or room air n=5–7) A separate group of hypoxic and normoxic dams were treated with N-acetylcysteine (500–600 mg/kg per day) in the drinking water for 10 days during the hypoxic period or during the age-matched gestational period in normoxic. Dams were euthanized on gestational day 63.	↓ fetal body weight with hypoxia. No effect of NAC on fetal body weight Oxidative stress in the fetal liver, resulting in cellular and nuclear damage with hypoxia. Alterations in cellular membrane integrity and aggregation of erythroid cells in fetal liver with hypoxia. ↑ fetal MDA levels with hypoxia.	N/A
[48] Tolcos, 2000. DOI: 10.1006/exnr.1999.7272	Aim: investigate the brain of offspring following chronic prenatal CO exposure during the last 60% of gestation, alone or in combination with acute hyperthermic stress.	Pregnant damswere exposed to 200 p.p.m CO for 10 h/day from gestational day ~24 until term (n = 11) or normal air (n = 10). At birth, offspring and dams were immediately removed from the chambers and raised in air for either 1 week (n=5 Control and n=7 CO-exposed dams) or 8 weeks (n=5 Control, n=4 CO-exposed dams). On postnatal day 4, all offspring were exposed to water at 23°C (normothermia) or 35°C (hyperthermia) for 75 mins. Offspring were assessed at 1 week or 8 weeks of age.	No effect on litter size or average birth weight No effect on offspring brain or body weight at 1 week or 8 weeks of age with prenatal CO exposure. No effect on structure of medulla or cerebellum in offspring with prenatal CO exposure. Prenatal CO exposure and postnatal hyperthermia resulted in lesions throughout the brain, in particular in the cerebral cortex, thalamus, and the medulla. ↑ GFAP-IR in the medulla with combined CO exposure and hyperthermia.	Information not provided
[40] Hodyl, 2007. DOI: 10.1016/j.bbr.2006.12.008	Aim: focus on functional differences in HPA efficacy, in the offspring of mothers exposed to endotoxin, at three developmental time points: at term, pre-weaning period and in adulthood.	Pregnant guinea pigs were administered endotoxin (Salmonella Enteritidis, 50 μg/kg) (n = 9) on gestational day 46, 48, 50 and 52, to mimic prolonged immune activation.	No differences in maternal weight on gestational day 46, 48, 50 and 52. No difference in litter size or fetal sex distribution. No difference in offspring weight from birth to adulthood. No effect on maternal serum cortisol concentrations at the time of caesarean section. ↓ fetal cortisol on gestational day 66. ↓ cortisol following exposure to the novel environment in offspring from endotoxin dams	Information not provided
[63] Wang, 2010. DOI: 10.1016/j.ijpharm.2010.05.030	Aim: report a model of chorioamnionitis and show the effective inhibition of bacterial growth by treatment with G4-PAMAM-OH.	Pregnant dams at gestational day 52 were inoculated intra-cervically with 150 CFU E. coli (n=11) to induce infection. Dendrimer G4-PAMAM-OH (500 μg) was injected into the cervix 5 min after E. coli inoculation (n=4). The E. coli inoculated dams without treatment were used as positive control (n=4). The dams without any treatment and inoculation were used as negative controls (n=3). 48 h after intervention, dams were euthanized.	150 CFU of E. coli was found to effectively induce the infection in the pregnant guinea pigs without leading to abortion of fetuses. 57.1% of the amniotic fluid samples from inoculated dams were positive with bacterial growth ↑ cytokines, especially TNFα and IL-6, in the placenta.	N/A
[69] Faralla, 2016. DOI:	Aim: Identify tissue-specific L. monocytogenes virulence determinants in vivo.	Pregnant dams (n=4) were injected with 109 CFU of the transposon mutant library. Bacteria surviving 24 h post injection was measured in maternal liver (n=4) and placenta (n=8).	Detailed histological examination of the maternal-fetal interface demonstrated inflammatory lesions with abundant bacteria and neutrophils	N/A
[64] Hensel, 2020. DOI: 10.1128/IAI.00204-20	Aim: to build upon previous intratracheal inoculation findings in nonpregnant guinea pigs by using intratracheal inoculation of pregnant guinea pigs to determine the effect upon reproductive success and to evaluate the pregnant guinea pig as an improved animal model for vaccine efficacy and safety.	Pregnant dams at approximately gestational day 20 to 25 (n=22) were allowed to acclimatize and implanted with a subcutaneous IPTT-300 microchip to monitor body temperature. At gestational day 30 to 35 days dams were inoculated with 107 CFU/50 B. melitensis 16M using an aerosolizer. At 7-day intervals groups of dams (n=4) were euthanized.	Dams develop fever ~14 to 18 days following inoculation. Stillbirth demonstrated by 21 days post inoculation in 25% of animals. Miscarriage continued to 24 days post inoculation in 20% guinea pigs and 30% offspring stillborn. Intracellular antigen within the chorioallantoic epithelium and cytotrophoblasts which became more severe and widely distributed by 24 days post inoculation. Character of the lesion in placenta shifted from small infiltrates to multifocal aggregates of histiocytes and foci of necrosis. Necrotizing placentitis colonization of the fetal liver and lung was detected in 23.5% of fetuses by 2 weeks post inoculation.	No - Fetal Demise
[66] Rollman, 2024. DOI: 10.1371/journal.ppat.1012515	Aim: to test how depleting T cells affects the course of primary cytomegalovirus infections.	Timed, semi allogenic pregnancies were established by breeding during postpartum estrus. Timed pregnancies were confirmed by progesterone at gestational day 21. At gestational day 35 days, pregnant dams were injected with 1×106 PFU of GPCMV and treated with 1.5 mg of α-CD4, α-CD8 IgG2b, or rat IgG (n=5/group). Blood and plasma were collected from the dams at 7 days post inoculation, when the antibody treatments were also repeated. The dams were necropsied either after euthanasia at 14 days post inoculation or after succumbing to GPCMV infection.	Severe illness and high rates of congenital infection were observed when helper CD4+ T cells were depleted. The depletion of killer CD8+ T cells did not affect the severity of disease or the rate of congenital infection but did increase the amount of virus that was detected in the placenta.	Maternal Loss
